# Artificial intelligence and virtual reality applied to the clinical care of women with schizophrenia: A systematic review.

**DOI:** 10.1192/j.eurpsy.2024.1527

**Published:** 2024-08-27

**Authors:** J. P. Paolini San Miguel, M. Natividad, M. V. Seeman, B. Palacios, A. Balagué, E. Román, N. Bagué, E. Izquierdo, H. Cachinero, J. A. Monreal, A. González Rodríguez

**Affiliations:** ^1^Mental Health, Mutua Terrassa University Hospital. University of Barcelona, Terrassa, Spain; ^2^Psychiatry, University of Toronto, Toronto, Canada; ^3^Clinical and Health Psychology, Autonomous University of the State of Morelos, Morelos, Mexico

## Abstract

**Introduction:**

Artificial intelligence (AI) and virtual reality (VR) are useful tools that can improve precision medicine and can prove useful in the clinical care of patients with psychosis.

**Objectives:**

Our aim was to determine whether AI and VR have been applied to the prediction of clinical response in women with schizophrenia.

**Methods:**

A systematic review was carried out in PubMed and Scopus from inception to September 2023 by using the PRISMA guidelines. Search terms: (“artificial intelligence” OR “intelligent support” OR “machine intelligence” OR “machine learning” OR “virtual reality” OR “intelligent agent” OR “neural networks” OR “virtual reality” OR “digital twins”) AND (“schizophrenia” OR “psychosis”) AND (“women” OR gender”). Inclusion criteria: 1)English, French, German or Spanish language, 2) reporting treatment response in schizophrenia (as long as information in women was included), and 3) including AI and VR techniques.

**Results:**

From a total of 320 abstracts initially screened (PubMed:182, Scopus:138), we selected 6 studies that met criteria.

- Prediction of treatment response. (1) Clinical information, genetic risk score and proxy methylation score have been shown to improve prediction models. (2) Graph-theory-based measures have been combined with machine learning.
- Therapeutic drug monitoring. (1) A machine learning model has been useful in predicting quetiapine blood concentrations.
- Pharmacovigilance. (1) Machine learning has connected prolactin levels and response in olanzapine-treated patients. (Zhu et al., 2022).
- Treatment-resistant schizophrenia (TRS). (1) Women with TRS have been found to receive clozapine less frequently than men (adjusted for sociodemographic, biological and clinical factors). (2) Statistical learning approach: Women have been found to respond better to clozapine than men.

**Conclusions:**

AI, including machine learning, show promising results in the prediction of treatment response in women with schizophrenia. As of yet, digital twins have not been investigated to test specific interventions or to personalize treatment in women with schizophrenia.

**Disclosure of Interest:**

None Declared

